# The application of nitric oxide to control biofouling of membrane bioreactors

**DOI:** 10.1111/1751-7915.12261

**Published:** 2015-03-06

**Authors:** Jinxue Luo, Jinsong Zhang, Robert J Barnes, Xiaohui Tan, Diane McDougald, Anthony G Fane, Guoqiang Zhuang, Staffan Kjelleberg, Yehuda Cohen, Scott A Rice

**Affiliations:** 1 Research Center for Eco-Environmental Sciences, Chinese Academy of SciencesBeijing, China; 2 School of Biological Sciences60 Nanyang Drive, SBS-01N-27, Singapore, 637551; 3 Advanced Environmental Biotechnology Centre, Nanyang Technological UniversitySingapore; 4 Singapore Membrane Technology Centre, Nanyang Environment and Water Research Institute, Nanyang Technological UniversitySingapore; 5 Singapore Centre on Environmental Life Sciences Engineering, Nanyang Technological UniversitySingapore; 6 Centre for Marine Bio-Innovation, School of Biotechnology and Biomolecular Sciences, The University of New South WalesSydney, NSW, Australia

## Abstract

A novel strategy to control membrane bioreactor (MBR) biofouling using the nitric oxide (NO) donor compound PROLI NONOate was examined. When the biofilm was pre-established on membranes at transmembrane pressure (TMP) of 88–90 kPa, backwashing of the membrane module with 80 μM PROLI NONOate for 45 min once daily for 37 days reduced the fouling resistance (R_f_) by 56%. Similarly, a daily, 1 h exposure of the membrane to 80 μM PROLI NONOate from the commencement of MBR operation for 85 days resulted in reduction of the TMP and R_f_ by 32.3% and 28.2%. The microbial community in the control MBR was observed to change from days 71 to 85, which correlates with the rapid TMP increase. Interestingly, NO-treated biofilms at 85 days had a higher similarity with the control biofilms at 71 days relative to the control biofilms at 85 days, indicating that the NO treatment delayed the development of biofilm bacterial community. Despite this difference, sequence analysis indicated that NO treatment did not result in a significant shift in the dominant fouling species. Confocal microscopy revealed that the biomass of biopolymers and microorganisms in biofilms were all reduced on the PROLI NONOate-treated membranes, where there were reductions of 37.7% for proteins and 66.7% for microbial cells, which correlates with the reduction in TMP. These results suggest that NO treatment could be a promising strategy to control biofouling in MBRs.

## Introduction

The membrane bioreactor (MBR) is a combined technology for the treatment of wastewater, which integrates the biological degradation of organics by activated sludge and separation of clean water from mixed liquor sludge suspension by membrane filtration into one system (Williams and Pirbazari, 2008). The difference between MBR-based water remediation and the traditional wastewater treatment process (WWTP) is in the separation of purified water from the sludge biomass, which is accomplished by using microfiltration or ultrafiltration membranes (MBR) instead of a settling tank (traditional WWTP). The MBR is advantageous because it reduces the treatment space, the production of sludge and the process time of water release, thus saving on capital expenditure and increasing the effluent quality (Van Nieuwenhuijzen *et al*., 2008).

However, biofouling remains a major limitation for the MBR technology (Wang *et al*., 2008). This biological phenomenon results from the formation of microbial biofilms on the membrane surface, which consists of cells bound by a self-produced matrix of extracellular polymeric substances (EPS), leading to plugging of the membrane pores (Meng *et al*., 2009). This process leads to a reduction of permeation flux when the MBR is operated under constant pressure conditions (Lee *et al*., 2008), or a rise of transmembrane pressure (TMP) when the system is operated under constant flux (Le Clech *et al*., 2003). Under both operational conditions, the fouling ultimately results in reduced productivity, increased treatment costs as well as a shorter lifespan of the membranes, limiting the general adoption of MBRs as a preferred wastewater treatment option (Meng *et al*., 2009).

A number of strategies have been tested to reduce biofouling in MBRs, such as modification of membrane surface properties (Tan and Obendorf, 2007), ultrasonic vibrations (Veerasamy *et al*., 2009) and chemical cleaning with acid or alkaline (Madaeni *et al*., 2009). Chemical cleaning is the most popular method in biofouling control, which can kill microbes and remove biofouling materials from the membranes (Lee *et al*., 2013). However, overuse of chemicals may damage the polymeric membrane structure (Puspitasari *et al*., 2010) and also pose an environmental and health risk (Estrela *et al*., 2002). Recently, a number of biological strategies have been regarded as promising methods to control the biofilm formation on membranes. For example, the quorum sensing (QS) inhibition agents, such as signal receptor antagonists and QS quenching enzymes, were reported to control biofilm formation in MBRs (Yeon *et al*., 2009a; Kim *et al*., 2012). Nitric oxide (NO) is an intracellular signalling molecule that has recently been shown to be involved in biofilm dispersal, inducing the transition from the biofilm mode of growth to the free swimming planktonic state (Barraud *et al*., 2006; 2014). NO induces biofilm dispersal by stimulating phosphodiesterase activity, resulting in the degradation of cyclic di-guanylate monophosphate (c-di-GMP), culminating in changes in gene expression that favour the planktonic mode of growth (Barraud *et al*., 2009b). The exogenous addition of NO, through the employment of low, sublethal concentrations of the NO donor compounds, such as 25–500 nM sodium nitroprusside and 5–80 μM PROLI NONOate, were shown to induce the dispersal of *Pseudomonas aeruginosa* biofilm and a range of other bacterial species and mixed species biofilms (Barraud *et al*., 2009b; Barnes *et al*., 2013). The difference in concentrations used in those studies primarily reflects the differences in NO donor chemistry and does not directly indicate actual NO concentrations achieved.

The aim of this research was to investigate the application of NO as a novel strategy to control membrane biofouling by complex microbial communities in an MBR system. The NO donor compound, PROLI NONOate, was applied to the membrane module at different biofouling stages (low and high TMP stages) to investigate its effect on the reduction of TMP and fouling resistance on the membrane.

## Results

### The effect of NO on established biofilms at high TMP stage

Data from previous experimental work showed that, during the biofouling process, the TMP increased in two distinct phases, where the TMP steadily increased from 3 to 15 kPa and a phase of rapid increase from 15 to 90 kPa. Membrane plugging caused by biomass from the activated sludge attaching to the hollow fibre (HF) membranes to form a biofilm has been proposed to be the primary reason for the rapid increase in the TMP (Chen *et al*., 2013). Since NO has been shown to reduce biofilms by inducing their dispersal in a non-toxic fashion, the effect of NO on the established MBR biofilms was studied by backwashing the NO donor compound PROLI NONOate into the membrane module in vitro once the MBR had reached the high TMP stage, 88–90 kPa. Two MBRs, operated in parallel, were used, where one was treated with the NO donor (MBR-1) and the second (MBR-2) served as the untreated control. During operation, the Mixed Liquor Suspended Solids (MLSS) and hydraulic retention time were maintained at approximately 2–4 g l^−1^ and 10 h respectively. The removal efficiency of the total organic carbon (TOC) was maintained at 95–98%.

**Fig 1:**
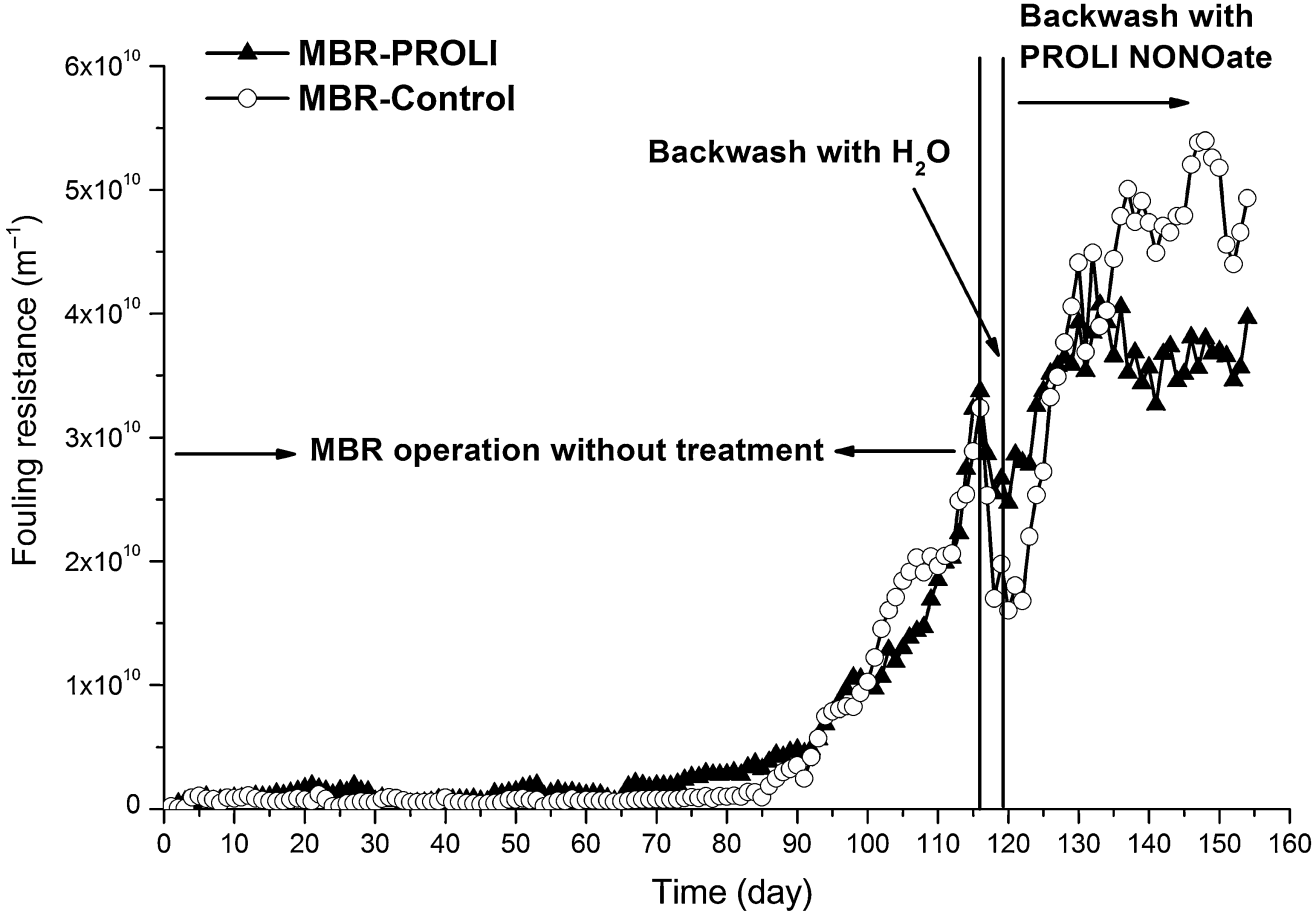
The effect of PROLI-NONOate backwashing treatment on pre-established biofilm at high TMP. Fouling was quantified by determining the resistance of the membrane to the passage of water. The vertical lines separate the experiment into three phases: normal MBR operation, backwashing with dH_2_O (distilled water) and backwashing with PROLI NONOate. The R_f_ values were determined after daily backwashing.

The MBR system, operated under a constant flux of 15 l m^−2^ h^−1^, reached the maximum TMP (88–90 kPa) after 116 days with a fouling resistance (R_f_) of 3.37 × 10^10^ m^−1^ for MBR-1 and 3.24 × 10^10^ m^−1^ for MBR-2 (Fig. 1) at which time, backwashing with NO was tested as a means to remove the existing biofilm on the HF membranes. After 2 days of backwashing with distilled water (dH_2_O), the R_f_ was reduced to 2.55 × 10^10^ m^−1^ for membrane MBR-1 and 1.7 × 10^10^ m^−1^ for membrane MBR-2 (Fig. 1). R_f_ is an indicator of the hydraulic resistance at the membrane and increases as a consequence of biofouling of the membrane (Martin *et al*., 2014). MBR-1, which had a slightly higher R_f_, was selected for the NO treatment and MBR-2 was used as the control module. During the operation, the TOC removal efficiency in the MBRs was within the expected range of 95–98%. Since the two modules had reached the maximum TMP achievable for the system, the effect of NO addition was monitored as a change in the resistance.

After 9 days of treatment (PROLI NONOate backwashing 45 min per day), the resistance increased for both the PROLI NONOate (NO)-treated membrane module and control membrane module, with values of 3.58 × 10^10^ m^−1^ and 3.49 × 10^10^ m^−1^ respectively (Fig. 1). However, the R_f_ of the NO-treated module increased at a lower rate than the control module, 1.4-fold increase versus 2.1-fold. After 10 days of treatment, the R_f_ for the control module was higher than the R_f_ for the NO-treated module. When the backwashing experiments were terminated at 155 days (37 days of treatment), the R_f_ of the NO-treated membrane module had increased to 3.97 × 10^10^ m^−1^, while the Rf for the control membrane module had increased to 4.93 × 10^10^ m^−1^ (Fig. 1). During the PROLI treatment, the TMP was not reduced and remained at the maximum level, 88–90 kPa (Fig. S1). However, compared with the first day of NO treatment, the R_f_ had increased by 1.42 × 10^10^ m^−1^ for the NO-treated membrane module and 3.23 × 10^10^ m^−1^ for the control membrane module, indicating that the R_f_ increase had been reduced by 56% due to the NO treatment. Therefore, the PROLI NONOate treatment could delay the increase of fouling resistance for a fouled MBR membrane.

### The effect of continuous NO addition on biofouling

The effect of NO on biofouling when NO donor was added from the beginning of operation of the MBR was also tested. In these experiments, the treated membrane module was exposed to a solution of the NO donor daily for 1 h. The control module was similarly rinsed in the same solution without the NO donor present. In these experiments, the treated membrane module was rinsed in a solution of the NO donor daily for 1 h from the start of the MBR operation. The control module was similarly rinsed in the same solution without the NO donor present. Since the membranes had no biofilm on their surface at the start of the experiment, the change in performance was determined by quantifying changes in both TMP and R_f_.

**Fig 2:**
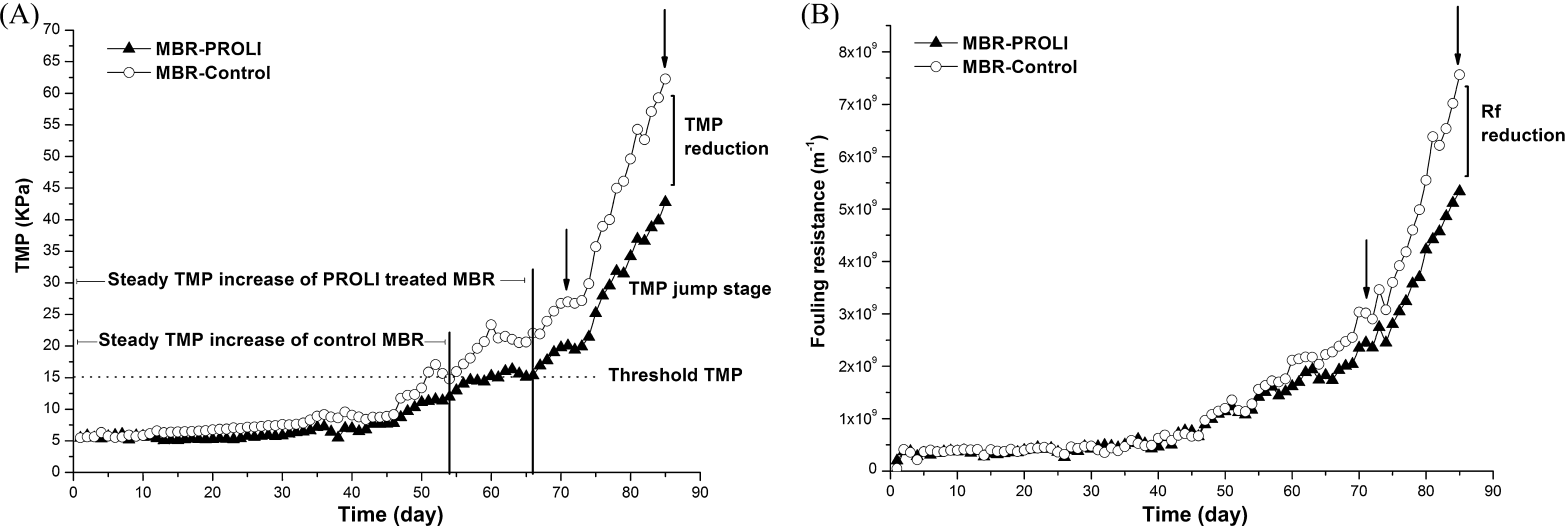
The effect of PROLI-NONOate treatment on the formation of biofilms from the commencement of MBR operation. The horizontal dashed line indicates the threshold TMP between the low pressure TMP stage and the TMP jump stage. The vertical lines show when PROLI-treated MBR and control MBR reached the TMP jump stage. The downward pointing arrows indicate the times at which membrane and sludge samples were collected and analysed to determine TMP (A) and R_f_ (B). The R_f_ values were determined after the daily PROLI NONOate treatment.

For the control membrane module, the system maintained operation in the low TMP stage (3–15 kPa) for 54 days before entering the TMP jump stage at which time the TMP increased rapidly. In comparison, the NO-treated membrane module was observed to remain in the low TMP stage for 66 days before entering the TMP jump phase (Fig. 2). The average rates of TMP increase were 0.24 kPa day^–1^ for the NO-treated module and 0.294 kPa day^–1^ for the control module in the steady TMP stage (3–15 kPa). When the experiment was terminated at 85 days, the TMP of the control membrane module had increased to 62 kPa while the NO-treated membrane module had only reached to 42 kPa (Fig. 2), representing a 32.3% reduction in TMP due to the NO treatment. The experiment was repeated and, due to time constraints, terminated at 34 days. In this experiment, the control TMP at the end of the experiment was 7 kPa and the PROLI-treated was 5.6 kPa, representing a 20% reduction in the TMP observed for the NO-treated module (Fig. S1). Thus, the NO treatment resulted in a slower TMP increase in MBR.

The R_f_ profiles for the NO-treated membrane module and control membrane module were similar during the first 46 days of operation. However, after 55 days, the R_f_ of the control module was higher than that of the NO-treated module (Fig. 2B). At 85 days, the R_f_ was 7.56 × 10^9^ m^−1^ for the control module and 5.43 × 10^9^ m^−1^ for the NO-treated module, representing a reduction of 28.2% in R_f_. This was consistent with the effect of PROLI NONOate treatment on the TMP increase.

### The effect of NO treatment on the biofilm bacterial community

**Fig 3:**
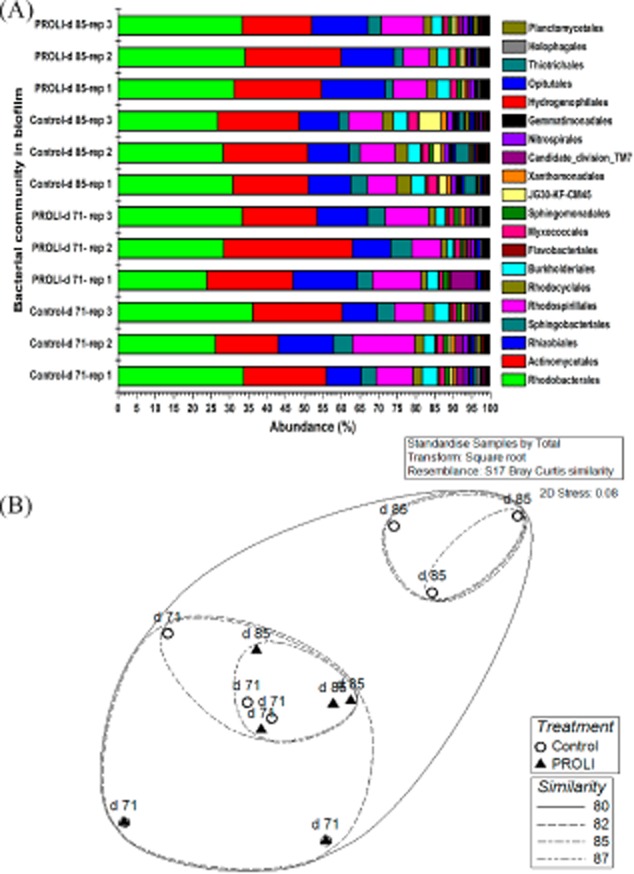
Bacterial communities on control and PROLI-NONOate-treated membranes. (A) The composition of bacterial communities at 71 days and 85 days. (B) The nonmetric multidimensional scaling (NMDS) plot for biofilm bacterial communities based on the Bray–Curtis similarity. ‘rep’ indicates the replicate sample. ‘PROLI’ represents the PROLI NONOate treatment.

To study changes in the microbial communities when NO was used to control fouling from the start of MBR operation, samples were collected at 71 days (20 kPa for NO treatment and 27 kPa for control) and 85 days (42 kPa for NO treatment and 62 kPa for control). The composition of the bacterial community was conserved in the treated and control biofilms on the 2 days tested (71 and 85 days), showing 80% Bray–Curtis similarity (Fig. 3 and Fig. S2). The dominant bacteria in the control and treated biofilms were similar, such as Rhodobacterales, Actinomycetales, Rhodospirillales, Rhizobiales and Sphingobacteriales (Fig. 3A). However, the bacterial community in the NO-treated biofilms at 85 days was closer to the control biofilm community at 71 days (85% similarity) relative to the control biofilm community at 85 days (80% similarity) (Fig. 3B).

**Fig 4:**
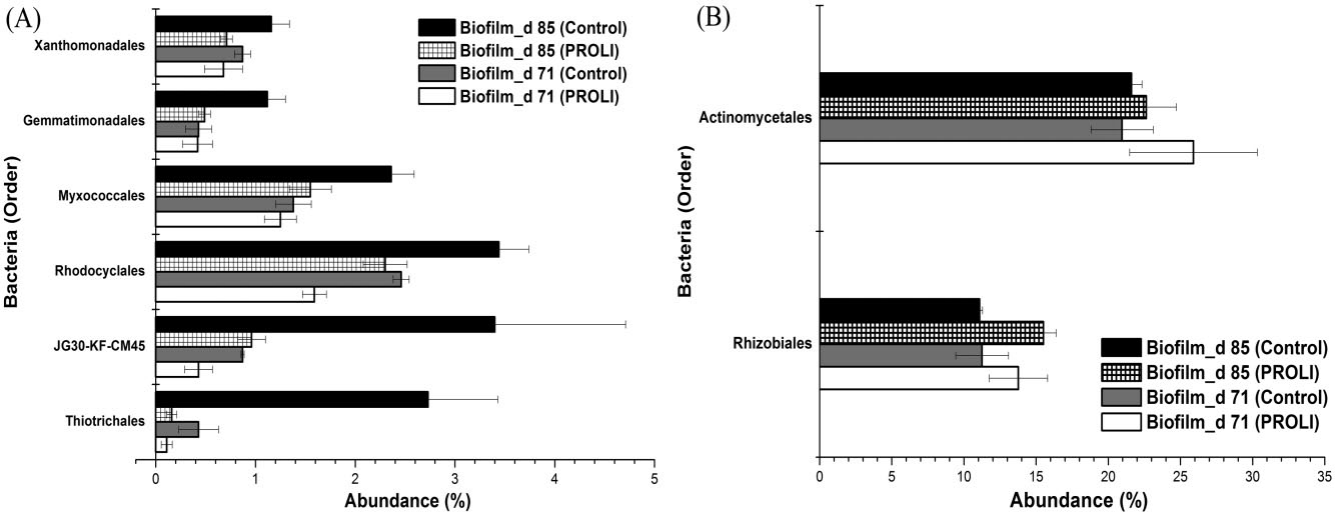
The change in MBR biofilm communities due to PROLI NONOate treatment. (A) Bacteria that have a lower abundance in the PROLI NONOate-treated biofilms relative to the control biofilms. (B) Bacteria have a higher abundance in the PROLI NONOate-treated biofilms relative to the control biofilms. The abundances were the average values of triplicate samples for the biofilms. The error bars are the standard errors of the mean (*n* = 3 replicate membrane samples).

There were some minor differences in the bacterial communities in the relative proportions of organisms between the control and treated samples. Five Orders of bacteria, including Thiotrichales, Gemmatimonadales, Xanthomonadales, Rhodocyclales and Myxococcales, had lower abundances in the NO-treated biofilms relative to the untreated control biofilms at both 71 and 85 days (Fig. 4). For example, at 85 days, the Thiotrichales accounted for 2.73% of the bacterial community on the control membrane compared with 0.16% on the NO-treated membranes. This trend was also observed for Gemmatimonadales and Xanthomonadales, which had abundances of 1.12% and 1.16% respectively in control biofilms at 85 days, in comparison to abundances of 0.49% and 0.71% respectively in NO-treated biofilms. These results indicate that these biofilm bacteria may be highly susceptible to dispersal and subsequent removal in the presence of NO.

Additionally, some bacteria, such as Rhizobiales and Actinomycetales, were found to have slightly higher abundances in the NO-treated biofilms in comparison to the control biofilms at both 71 and 85 days (Fig. 4). The Rhizobiales accounted for 13.77% and 15.51% respectively in the NO-treated biofilm community at 71 and 85 days, while their abundances were 11.26% and 11.08% in the control biofilms. Similarly, the abundance of Actinomycetales was also higher in the NO-treated biofilms (25.91% at 71 days and 22.64% at 85 days) than in the control biofilms (20.96% at 71 days and 21.61% at 85 days). This indicated the Rhizobiales and Actinomycetales may not be dispersed by NO as effectively as other bacteria, or that these bacterial may be able to utilize NO for biofilm formation, resulting in relative higher abundances for them in the NO-treated biofilm community.

### The effect of NO treatment on the biofilm fungal community

**Fig 5:**
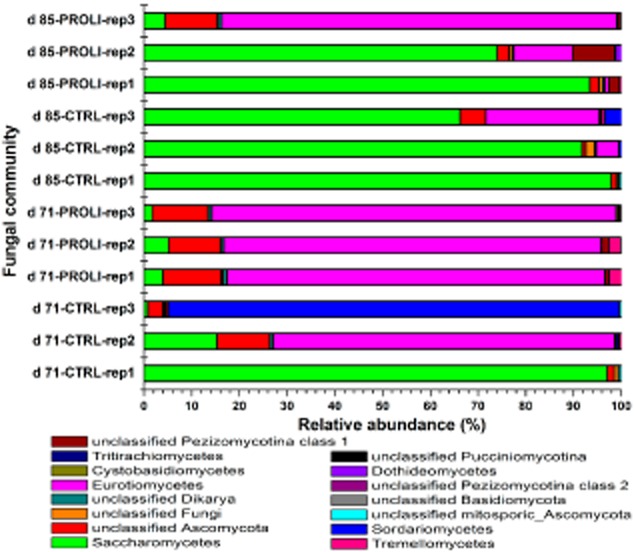
The fungal community compositions for PROLI NONOate-treated and control biofilms after 71 and 85 days of MBR operation. ‘CTRL’ and ‘PROLI’ represent the control and PROLI NONOate-treated biofilms. ‘rep’ indicates the replicate sample.

**Fig 6:**
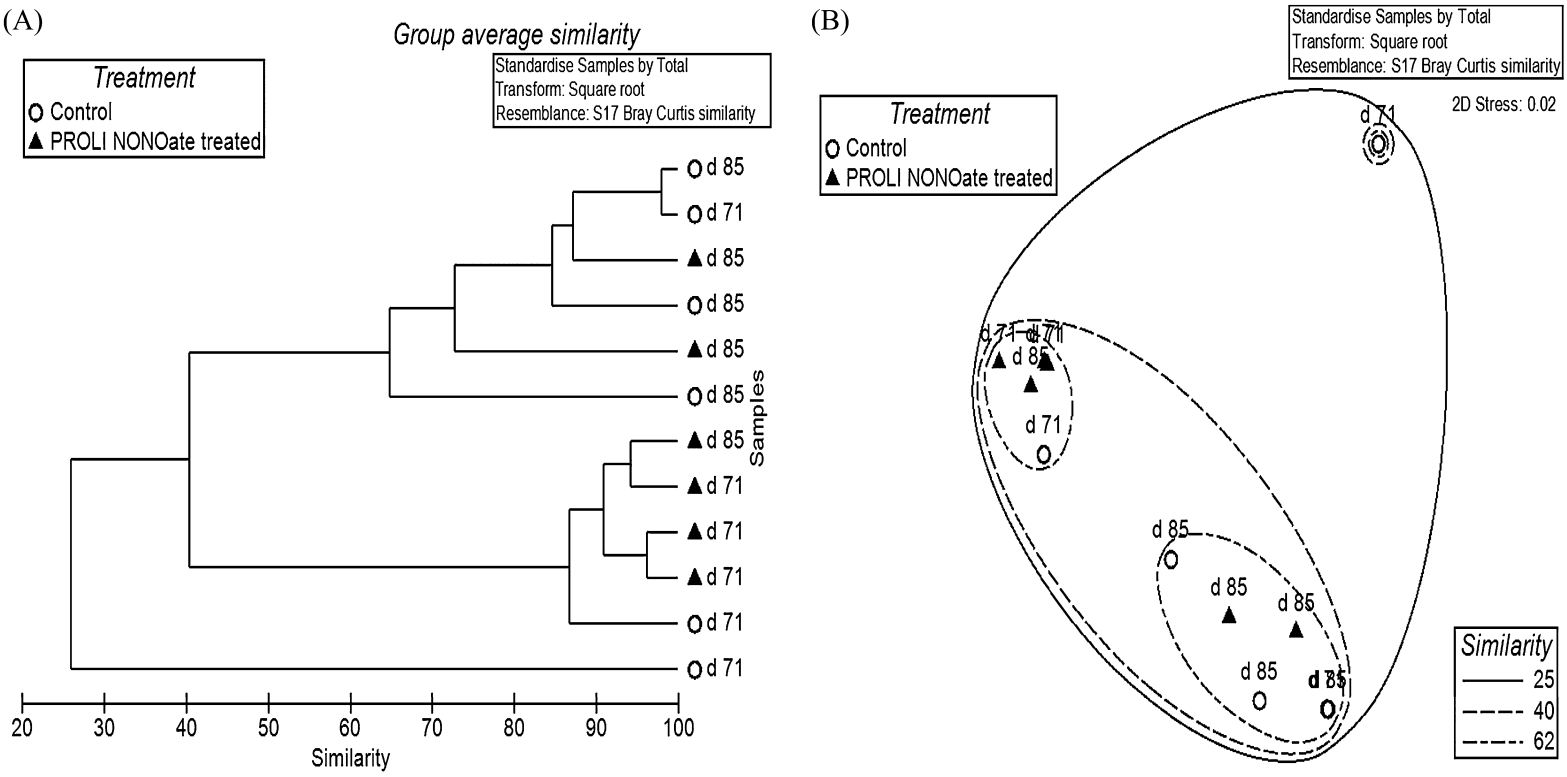
Comparison of fungal communities for the PROLI NONOate-treated biofilms and control biofilms. (A) The clustering tree for the fungal communities. (B) The nonmetric multidimensional scaling (NMDS) plot for the fungal communities.

**Fig 7:**
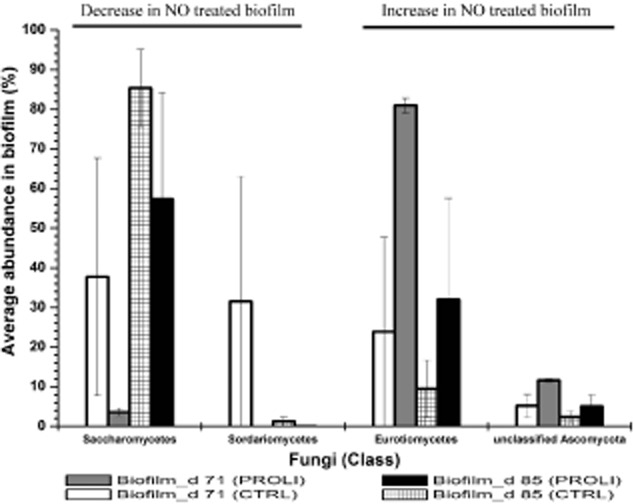
The variation of fungi in MBR biofilms due to PROLI NONOate treatment. The left two fungi were decreased in abundance in the PROLI NONOate-treated biofilms. The right two fungi were increased in abundance in the PROLI NONOate-treated biofilms. The abundances are the average values of triplicate samples for the biofilms. The error bars are the standard errors of the mean (*n* = 3 replicate membrane samples).

The biofilm fungal community was also compared for the NO-treated and control biofilms at 71 and 85 days (Fig. 5). The dominant fungal groups were conserved in the two types of biofilms and included Eurotiomycetes, Saccharomycetes, Sordariomycetes and unclassified Ascomycota. However, distinct from the bacterial community, the fungal communities distributed unevenly in the replicated biofilm samples, resulting in the lower similarity (32.73% at 71 days and 60.9% at 85 days) between the NO-treated and control biofilms (Fig. 6 ). Examining the average abundance, the Saccharomycetes and Sordariomycetes became less abundant in the NO-treated biofilms while the Eurotiomycetes and unclassified Ascomycota increased the abundance in the NO-treated biofilms (Fig. 7). Specifically, the Saccharomycetes accounted for 37.7% and 85.4% of the fungal community in control biofilms at 71 days and 85 days respectively but decreased in abundance to 3.6% (71 days) and 57.3% (85 days) in the NO-treated biofilm fungal communities. The Sordariomycetes had the abundance of 31.6% (71 days) and 1.3% (85 days) in the control biofilms. However, this fungal class was not detected in the NO-treated biofilm at 71 days and had a quite low abundance (0.02%) in the NO-treated biofilms at 85 days. Given that there was a significant reduction in abundance of the Sordariomycetes for the untreated biofilm from 71 days to 85 days, it is difficult to be certain that the reduced percent composition for the NO was a consequence of the NO treatment or whether it was due to other factors. For the other two Classes of fungi, the Eurotiomycetes accounted for 23.92% and 9.5% of the fungal community in the control biofilms at 71 and 85 days respectively, compared with 81% and 31.98% in the NO-treated biofilms. One unclassified class of Ascomycota was also present in the biofilm at a higher abundance in the NO-treated biofilms (11.6% at 71 days, 5.11% at 85 days) relative to the control biofilms (5.21 % at 71 days, 2.41 % at 85 days).

### NO treatment results in a reduction of biofilm constituents

**Fig 8:**
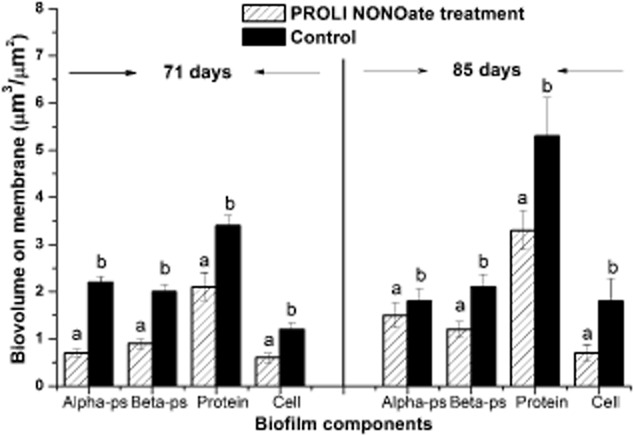
The biovolumes of biofilm components on membranes as visualized by confocal microscopy for the untreated control and PROLI NONOate-treated MBRs. ‘Alpha-ps’ and ‘Beta-ps’ represent the Alpha-polysaccharides and Beta-polysaccharides respectively. The error bars are the standard error of the mean (*n* = 30). For each PROLI NONOate-treated and control biofilm component at the same day, the bars at different letters (A, B) are significantly different at *P* < 0.05 (*t*-test).

In order to determine the effect of NO treatment on the biomass of biofilm, the different biofilm components were quantified by the confocal laser scanning microscopy (CLSM) analysis at the 2 days tested. Four components, including proteins, α-polysaccharides, β-polysaccharides and total cells, were studied. The biovolume of each biofilm component in the NO-treated module was observed to be lower than the corresponding components in the control MBR (Fig. S3). For example, at 71 days, the biovolume of the proteins was 2.1 μm^3^ μm^−2^ on the NO-treated membrane and 3.4 μm^3^ μm^−2^ on the control membrane (Fig. 8), representing a 38.2% reduction as a consequence of NO treatment. Similarly, the biovolumes for the α-polysaccharides, β-polysaccharides and microbial cells were reduced by 68.1%, 55% and 50% respectively on the NO-treated membranes at 71 days. At 85 days, although the biovolumes of each biofilm component had increased from 71 days in both control and NO-treated modules, they were reduced on the NO-treated membranes. For example, the α-polysaccharide biovolume was 1.8 μm^3^ μm^−2^ for the control biofilms compared with 1.5 μm^3^ μm^−2^ in the NO-treated biofilms. The greatest differences observed at 85 days were for the protein and microbial cells, which were 5.3 μm^3^ μm^−2^ and 1.8 μm^3^ μm^−2^ respectively on control membranes compared with 3.3 and 0.7 μm^3^ μm^−2^ on the PROLI NONOate-treated membranes (Fig. 8). Thus, there was a 37.7% reduction in protein and a 66.7% reduction in microorganisms associated with the NO treatment on day 85. Moreover, the amount of DNA extracted on 71 days was 2.7 μg cm^−2^ for the control biofilms and 2.3 μg cm^–2^ for the NO-treated biofilms. At 85 days, 4.2 μg cm^−2^ of DNA was extracted from the control membrane while 3.98 μg cm^−2^ of DNA was extracted from the NO-treated membrane (data not shown). This also suggested that the number of microorganisms on membranes was reduced by NO treatment and was consistent with the image-based assessment of the total biovolume of microorganisms.

## Discussion

### NO treatment delayed the increase of TMP and fouling resistance

Membrane cleaning with chemical agents, such as sodium hypochlorite, is the most commonly used method to control biofouling (Lee *et al*., 2013). However, the application of sodium hypochlorite may adversely affect the structure, and hence function, of polymeric membranes (Puspitasari *et al*., 2010) and the biological function of activated sludge (Lee *et al*., 2013). It has been demonstrated that sublethal concentrations of NO can induce biofilm dispersal in a range of bacteria (Barraud *et al*., 2006; Hetrick *et al*., 2009; Barnes *et al*., 2013). In this study, NO treatment, utilizing the NO donor PROLI NONOate, was shown to reduce the growth of biofilms formed by mixed species microbial communities in a MBR system, decreasing membrane fouling. After backwashing, established biofilms treated with NO for 37 days showed a 56% reduction of R_f_ relative to the control membrane module. This indicated that backwashing with NO could partially reduce the growth of established biofilms in MBR. Similarly, exposing the membrane to NO from the start of the experiment resulted in a 32.3% reduction of TMP and a 28.2% reduction of R_f_, indicating that the PROLI NONOate treatment could also delay the construction of the new biofilms on membrane. When repeated, the TMP increase was again delayed by 20% after NO treatment for 34 days, showing that effect of NO on TMP and biofouling was reproducible. The delay in TMP increase was supported by the image-based data, which showed that NO treatment resulted in a reduction of biofilm biomass, for both cells (66.7% reduction) as well as macromolecules (e.g. 37.7% reduction for proteins). This finding was consistent with previous results, where a 30% reduction of total microbial biovolume was observed when the biofilms were treated with 80 μM PROLI NONOate for 1 h (Barnes *et al*., 2013). In combination, these results demonstrate that the proof-of-principle that NO can be used reduce the growth of biofilms in a MBR, delaying the TMP jump and hence increasing the operational time of the MBR.

In previous studies, biological methods based on the molecular signalling have already been demonstrated to control biofouling in MBR systems. For example, it was reported that the Porcine kidney acylase I, which is a quenching enzyme of *N*-acyl homoserine lactone autoinducers, could prevent biofouling (approximately 26–46% reduction in TMP) in MBR (Yeon *et al*., 2009a,b; Kim *et al*., 2013). In this study, NO released from the PROLI NONOate also acts as a biofilm dispersal signal molecule and involves c-di-GMP-based signal transduction (Barraud *et al*., 2009a). Thus, it is concluded that the signalling-based biological strategies, e.g. NO or anti-QS approaches, may be promising in fouling control in MBRs. However, the NO-based biofouling prevention strategy was not as efficient as the commonly used chemical cleaning strategy, which was reported that treatment with the combination of 3000 mg l^−1^ NaClO and 500 mg l^−1^ NaOH reduced 81.8% of TMP (Li *et al*., 2011) and treatment with 1000 mg l^−1^ citric acid recovered 90% of the membrane permeability in MBR (Yan *et al*., 2012). Thus, further experiments are required to improve the biofouling prevention efficiency of NO-based biological strategy, such as optimizing the dosing concentration and regimen of NO-releasing donor or choosing a more stable NO-releasing chemical. Additionally, we have shown that NO can increase the sensitivity of the biofilm to standard cleaning or killing agents, such as hydrogen peroxide or surfactants (Barraud *et al*., 2006). Therefore, one strategy that might be successful is to induce biofilm dispersal using NO and to include a standard cleaning agent, such as chlorine or caustic treatment to better remove the biofilm.

### Effect of NO treatment on the microbial community in biofilm

While the NO treatment was able to delay the increase in biofilm biomass and the associated TMP rise, it was not able to prevent it completely. Characterization of the biofilm communities indicated that the bacterial and fungal communities in the treated and control biofilms were of high similarity, where the dominant bacteria were Rhodobacterales, Actinomycetales, Rhodospirillales, Rhizobiales and Sphingobacteriales and the dominant fungi were Eurotiomycetes, Saccharomycetes and Sordariomycetes. Based on the previous microbial community analysis (data not shown), bacteria including Actinomycetales, Rhodospirillales, Rhizobiales and Sphingobacteriales were the dominant biofilm forming microorganisms at high TMP. In previous studies, the Actinomycetales strains were also isolated from cave biofilms and biofilms on MBR membranes (Kim *et al*., 2005; Jurado *et al*., 2009). The Rhodospirillales and Rhizobiales have been reported in the biofilms of urban drinking water distribution system (Liu *et al*., 2012). The Saccharomycetes, which were the significant sludge community compositions in wastewater treatment systems (Yang *et al*., 2011), have also been proposed to be important in the biofouling process of MBRs and groundwater treatment facilities (Gillings *et al*., 2013; Xia *et al*., 2010). Thus, the treatment with 80 μM PROLI NONOate in this study did not significantly alter the microbial biofilm community composition. However, comparison of the community similarity suggested that the biofilm bacterial community observed for the NO-treated biofilms at 85 days was slightly more similar to the control biofilms at 71 days rather than the control biofilms at 85 days, indicating the development of bacterial community may be delayed by the NO treatment. While it is tempting to speculate that the NO treatment delayed the establishment of a community associated with the maximum TMP, further work is needed to more accurately track the change in community composition across the TMP curve to address this. It should be noted that these data represent relative abundances and not absolute numbers of community members. Thus, it is not possible to determine if the changes in community abundance represent an increase in a specific Operational Taxonomic Unit (OTU) or the lack of dispersal for that organism relative to the other community members.

While the overall biofilm community composition was not strikingly different in the control and treated MBRs, there were still some subtle differences in both the bacterial and fungal communities after NO treatment. The bacterial orders of Thiotrichales, Gemmatimonadales, Xanthomonadales, Rhodocyclales and Myxococcales and fungal classes of Saccharomycetes and Sordariomycetes were reduced in abundance in the PROLI NONOate-treated biofilms. In previous works, the Gemmatimonadales were reported to preferentially form the base structure of biofilm (Besemer *et al*., 2007). The Xanthomonadales and Rhodocyclales had been identified as biofilm constructors on polyvinyl chloride membranes in MBR (Xia *et al*., 2010). The Saccharomycetes and Sordariomycetes were primary fungal compositions of biofilm in kitchen and bathroom sinks (Adams *et al*., 2013). Interestingly, these organisms were reduced in abundance in the NO-treated biofilms. This may suggest that these organisms are particularly sensitive to NO-mediated dispersal and their removal from the biofilm may be important in the observed delay in TMP increase. Therefore, it will be of interest to quantify those specific organisms in future experiments by fluorescence *in situ* hybridization or quantitative PCR to determine their overall relationship to membrane fouling and the TMP rise.

Some bacteria showed an increased abundance in the NO-treated biofilm, such as Rhizobiales and Actinomycetales. Although the NO has been reported to induce dispersal of bacteria from both single and mixed species biofilms (Barraud *et al*., 2006; 2009b; Barnes *et al*., 2013), it has also been shown that some bacteria do not disperse in the presence of NO (Barnes *et al*., 2013) and NO can actually stimulate the biofilm formation of some bacteria, e.g. *Azospirillum brasilense* (Arruebarrena Di Palma *et al*., 2013) and *Shewanella oneidensis* (Plate and Marletta, 2012). This may explain why these bacteria had a relative higher abundance in PROLI NONOate-treated biofilm. Further investigation is required to determine the specific mechanism for them to have increased abundance in NO-treated biofilm.

In conclusion, the NO donor compound PROLI NONOate showed the potential to control membrane biofouling in MBRs through reducing the production of macromolecules in EPS, delaying the succession of the microbial community and selectively dispersing some microbial groups. While biofouling was not completely prevented, these results are significant as the microbial community of the MBR is highly diverse, comprised here of approximately 103 orders of bacteria and 17 classes of fungi. Further work aimed at optimizing the delivery of NO may improve the overall efficacy of NO in biofouling control. Additionally, alternative NO donor compounds, which exhibit different NO release kinetics, may be better suited for the high organic content environment of the MBR. It has been shown previously that biofilms exposed to NO donor compounds were more susceptible to antimicrobial agents and removal from surfaces with surfactants (Barraud *et al*., 2006). Therefore, NO treatment may be best complemented with traditional biofilm cleaning protocols, e.g. bleaching with low concentration of chlorine, to synergistically remove the biofilms and hence, alleviate the fouling problem.

## Experimental procedures

### MBR set-up

A laboratory scale submerged MBR was operated to treat artificial synthetic wastewater, composed of 320 mg l^−1^ glucose, 60 mg l^−1^ beef extract, 80 mg l^−1^ peptone, 7 mg l^−1^ KH_2_PO_4_, 14 mg l^−1^ MgSO_4_●7H_2_O, 7.3 mg l^−1^ FeSO_4_●7H_2_O and 90 mg l^−1^ sodium acetate. The TOC was 200 mg l^−1^. The MBR system was composed of an anoxic sludge tank and an aerobic sludge tank containing two separate membrane modules (Fig. S4). The membrane modules were made with HF Polyvinylidene fluoride (PVDF) membranes (ZeeWeed, GE) and were assembled as a ‘curtain’ style module. The area of membrane was 565 cm^2^ for each membrane modules. For the membrane pieces, one end (the free end) was sealed and hung down into the sludge tank. The other ends of the HF membranes were open and sealed into a chamber that was linked to the suction pump.

Before the experiment, fresh activated sludge was collected from the Ulu Pandan wastewater treatment plant in Singapore and acclimated in artificial synthetic wastewater for 60 days before the start of the experiment. During MBR operation, the synthetic wastewater from the feedwater tank passed through the anoxic tank and aerobic tank and was degraded by the sludge biomass. The wastewater was then recycled from the aerobic tank to the anoxic tank (1.2 l h^−1^) to be further degraded. The purified water was subsequently separated from the sludge by the membrane module. The two modules were run with a constant flux of 15 l m^−2^ h^−1^. The aeration speed in the aerobic tank was 5 l min^−1^. The MLSS were maintained at 2–4 g l^−1^. The hydraulic and sludge retention times for the MBR were maintained at approximately 10 h and 25 days respectively. The MBR was run at a room temperature (25–26°C). The parameters, such as membrane flux, TMP, pH, dissolved oxygen and temperature, were monitored and automatically recorded using a data logger and computer. The TOC of the influent and permeate was measured using a multi N/C® 2100s (AnalytikJena).

### Application of the NO donor PROLI NONOate to membrane

The effect of NO on membrane biofouling was tested using two distinct approaches. The first approach was to immerse the membrane module into the solution of NO donor compound PROLI NONOate (Cayman Chemicals) from the beginning of the experiment. Another approach was to backwash the membrane module with PROLI NONOate solution after the TMP had increased to 88–90 kPa. All treatments were operated in separated tanks outside of the MBR sludge tank to ensure there was no effect of PROLI NONOate on the sludge microbial community.

For the continuous addition of the PROLI NONOate from the beginning of the MBR operation, the treatment was performed on the membrane modules every 24 h. The NO-treated module and control module were taken out from the sludge tank and treated in two separate beakers. For the NO-treated membrane module, the membrane was immersed in 80 μM PROLI NONOate in diluted synthetic feed water (TOC of 10 mg l^−1^, pH 8.5–9.0) for 1 h. Dosing concentrations were based on previously published work that used MBR isolates to determine effective concentrations (Barnes *et al*., 2013). For the control module, the membrane was immersed under the same conditions but without PROLI NONOate. After treatment, the modules were returned to the MBR and operation was continued. The TMP and flux (Jw) were measured immediately in dH_2_O at room temperature before and after the NO treatment. The hydraulic resistance through the membrane during filtration was calculated using the TMP and flux in pure water based on the formula R_f_ = (ΔP − Δπ)/μ Jw − R_m_ (Chen *et al*., 2012). The pressure differential (ΔP) and Jw are the measured TMP and flux. The osmotic pressure differential (Δπ) was 0 for pure water and μ is the viscosity of water at 25–26°C. R_m_ is the resistance for the clean membrane module, which was 1.5 × 10^8^ m^−1^ for the PROLI-treated membrane module and 1.1 × 10^8^ m^−1^ for control membrane module respectively.

To test the ability of the NO donor to remove an established fouling layer, the MBR system was operated until both membrane modules had reached the maximum TMP (85–90 kPa) after which time the PROLI NONOate was added. For treatment, the membrane modules were removed from the aerobic sludge tank and immersed into different beakers, which were filled with 500 ml diluted synthetic feed water (TOC of 10 mg l^−1^, pH 7). For the NO-treated membrane module (45 min exposure to NO per day), one backwashing pump was used to deliver 80 μM PROLI NONOate stock solution, which was dissolved in a 10 mM NaOH solution to prevent the spontaneous release of NO before it reached the membrane. Another backwashing pump, referred to here as the neutralizing pump, was used to deliver a 2.5 mM HCl solution. The ratio of flow rate for the PROLI NONOate backwashing pump and HCl pump was 1:4. The two solutions were mixed directly before they entered the membrane module to adjust the pH to be approximately 7 (± 1) and to hence ensure there was no effect of the NaOH or HCl on the biofouling of the membrane. These flow rates and concentrations were verified to produce a neutral pH (data not shown), which allows for NO release from the PROLI NONOate donor. The untreated control module was treated in the same way, but without PROLI NONOate. The total flux for the backwashing was 30 l m^−2^ h^−1^ for both the PROLI-treated and control modules, which was twice the suction flux during the MBR operation.

### Fluorescent staining and CLSM observation

Membrane pieces were collected to analyse microbial biomass by CLSM at two time points, 71 and 85 days. Three HF membranes were cut from the free ends of membrane module for each sampling point, immersed in 20 μM SYTO 63 red fluorescent nucleic acid stain solution (Molecular Probes, Invitrogen) and incubated at room temperature in the dark for 30 min to stain the DNA in the microbial cells. The membranes were then removed from the SYTO 63 staining solution and soaked in 500 μl fluorescein-5-isothiocyanate (FITC ‘Isomer I’, Molecular Probes, Invitrogen) staining solution (1 mg ml^−1^) for 1 h to image proteins in the biofilm. The membranes were then immersed in freshly prepared 0.2 mg ml^−1^ concanavalin A-tetramethylrhodamine (ConA) (Molecular Probes, Invitrogen) staining solution for 30 min to stain the α-mannopyranosyl and α-glucopyranosyl sugar residues. Finally, the membranes were stained with 1 g l^−1^ calcofluor white solution (Sigma) for 30 min to label the β-D-glucopyranose polysaccharides (McSwain *et al*., 2005). All staining procedures were performed at 25–26°C in the dark. After staining, the samples were immersed into phosphate-buffered saline for 10 min, twice to remove excess stain.

The stained membrane samples were put onto glass slides for imaging using an inverted CLSM (LSM 710, Carl Zeiss). Four channels were used to image the samples. The calcofluor white was excited at 405 nm and detected at 410–480 nm. The wavelengths used for FITC were 488 nm for excitation and 500–540 nm for emission. The tetramethylrhodamine-conjugated concanavalin A was detected via excitation at 543 nm and emission at 550–600 nm. The SYTO 63 was excited at 633 nm and captured at 650–700 nm (Chen *et al*., 2007). At every time point, three pieces of membrane were collected to stain. A total of 9–15 images were captured for the triplicate membrane samples to analyse. The quantitative analysis of the three-dimensional (3D) CLSM images performed using IMARIS (Version 7.3.1, Bitplane) software. The 3D structure of each biofouling component was reconstructed by the ‘surface’ function in IMARIS. The threshold of fluorescent intensity was adjusted to remove the pixels of background. The biovolume of the fluorescent pixels in each fluorescent channel was calculated by the command ‘statistics’. Finally, the biovolume of each biofouling component was standardized as the volume (μm^3^) of the specific component per membrane surface area (μm^2^). The significance of difference in biofouling components between the PROLI NONOate-treated and control biofilm was calculated by *t*-test in SPSS (version 16.0). Comparisons were considered significantly different at *P* < 0.05.

### DNA extraction

DNA from the biofouling community growing on the HF membranes as well as from the activated sludge in the bulk solution was extracted by a modified CTAB-Polyethylene glycol protocol (Paithankar and Prasad, 1991; Griffiths *et al*., 2000) at two time points, 71 and 85 days. For each sample point, three independent sludge samples or HF were collected for replicates. The HF membrane pieces were cut into small pieces and put into microfuge tubes containing lysing matrix (MP Biomedicals). Subsequently, 0.5 ml of 5% CTAB lysis solution and 0.5 ml phenol/chloroform/isoamyl alcohol (25:24:1) were added to the tubes. The tubes were placed in a Fast-Prep bead beater (FastPrep-24, M.P. Biomedicals) and shaken using speed setting 5.5 for 30 s. Afterwards, the tubes were centrifuged at 17 000 *g* for 5 min. The top aqueous layer was transferred to a clean 2 ml tube, RNase was added at a final concentration of 10 μg ml^−1^ and the sample was incubated at 37°C for 30 min. After RNA digestion, 0.5 ml chloroform/isoamyl alcohol (24:1) was added, the samples were vortexed briefly and centrifuged at 17 000 *g* for 5 min. The top aqueous layer was transferred into clean 2 ml tubes and mixed well with two volumes of a 30% PEG solution and incubated at 4°C overnight to precipitate the DNA. The following day, the samples were centrifuged at 17 000 *g* for 15 min and the supernatant was discarded. The DNA pellets were washed with 70% ice-cold ethanol three times and air dried. The DNA pellets were dissolved in DNase and RNase-free distilled water and the concentration was quantified using a NanoDrop spectrophotometer (Thermo Scientific). The aqueous DNA samples were stored at −80°C.

### Pyrosequencing and processing of sequence data

The DNA was sequenced using the ‘454’ pyrosequencing platform (Research and Testing Laboratory, TX, USA) targeting bacterial and fungal communities (Handl *et al*., 2011). The primers selected for the bacterial PCR were Gray28F (5′-GAGTTTGATCNTGGCTCAG-3′) and Gray519R (5′-GTNTTACNGCGGCKGCTG-3′) (Baker *et al*., 2003). The primers selected for the fungal PCR were forward funSSUF (5′-TGGAGGGCAAGTCTGGTG-3′) and reverse funSSUR (5′-TCGGCATAGTTTATGGTTAAG-3′) (Foster *et al*., 2013). The number of reads for every sample was approximately 3000.

The pyrosequencing data were processed using MOTHUR based on the Costello analysis pipeline (Costello *et al*., 2009; Schloss *et al*., 2009). The sequences were sorted by the barcodes to generate groups of data for the samples. The sequence packets were trimmed and barcodes and primers were removed. Sequences that had poor quality (below the quality score of 25) were removed from the dataset and the size of data packets was reduced to facilitate the analysis through the process of ‘unique’. Chimeric sequences were identified and removed using ‘chimera.slayer’. The reference was set to be self and the sequences were aligned with the SILVA bacterial 16S and eukaryotic 18S rRNA sequence databases and assigned to taxonomic groups based on the SILVA bacterial and eukaryotes taxonomic reference (Pruesse *et al*., 2007). The criteria for the sequence classification (identity to the reference sequence) were: species (> 97%), genus (94–97%), family (90–94%), order (85–90%), class (80–85%) and phylum (75–80%) (Lim *et al*., 2012). Sequences with similarities below these criteria were classified into unidentified groups for each taxonomic rank. Finally, a shared phylotype file was generated through the command ‘make.shared’ for all the samples. The coverage of phylotypes was calculated based on the phylotypes acquired and the number of sequences pooled. The relationship between the phylotypes and sequences was calculated by MOTHUR using the command of ‘summary.single’. All raw DNA sequences were deposited in the GenBank Sequence Read Archive. The accession numbers for the bacterial sequences are SRR1066760–SRR1066769, SRR1066771–SRR1066774, SRR1066776, SRR1066777, SRR1066780, SRR1066783, SRR1066784, SRR1066787, SRR1066788. The accession numbers for the fungal sequence are SRR1067677–SRR1067697.

### Phylotype-based community analysis

Phylogenetic trees and nonmetric multidimensional scaling plots were created based on the Bray–Curtis similarity of phylotype compositions in the different groups (Clarke, 1993). The similarity or dissimilarity between the samples or groups was calculated by ‘SIMPER’ analysis (PRIMER-E, UK) (Clarke, 1993). The contributions of the phylotypes to the similarity or dissimilarity were calculated based on the relative abundances of phylotypes between the samples. The fungi or bacteria which contributed 1% or more to the similarity or dissimilarity were considered to be the key organisms influencing the composition of the community.

## Supporting Information

**Fig. S1.** The TMP profile for the PROLI NONOate-treated and control membrane module. The TMP values are the daily average transmembrane pressures. The vertical dashed line splits the TMP profile into the low pressure phase (left side) and the rapid TMP increase phase (right side). **Fig. S2.** The clustering tree of the bacterial communities for the PROLI NONOate-treated and control biofilms at 71 and 85 days. The clustering tree was constructed based on the average Bray–Curtis similarity. The labels ‘control’ and ‘PROLI’ represent the control biofilms and PROLI NONOate-treated biofilms respectively. **Fig. S3. ** Representative 3D confocal laser scanning microscopic (CLSM) images of biofilm matrix components and cells on the PROLI NONOate-treated and control membranes. The α-polysaccharides (A) are shown in red, the β-polysaccharides (B) are shown in blue, the proteins (C) are shown in green and the microorganisms (D) are shown in purple. All images are top down projects of 3D reconstructions of the biofilm. The total magnification for the images was 630×. **Fig. S4.** Schematic drawing of the internal submerged MBR.
