# Supplementary material for: The application of nitric oxide to control biofouling of membrane bioreactors
Source: Microb Biotechnol. 2015 Mar 6;8(3):549–60. doi: 10.1111/1751-7915.12261 (PMC4408187; doi:10.1111/1751-7915.12261)
Supplement: Supplementary file 1 [file mbt20008-0549-sd1.docx]

**Supplementary information**


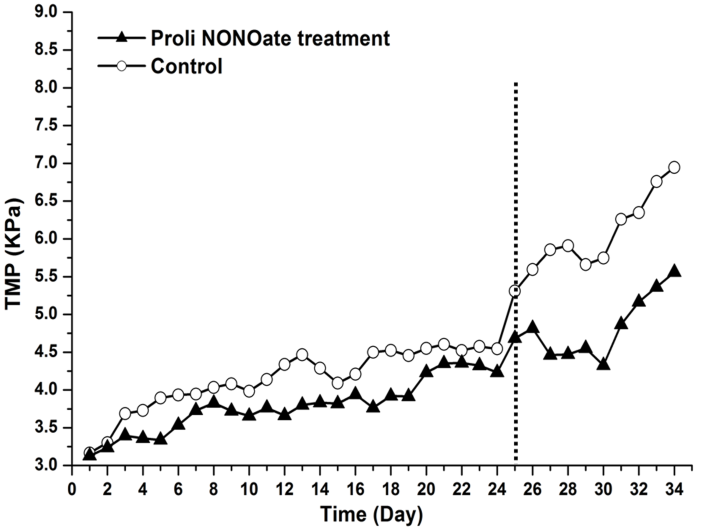


**Fig. S1.** The TMP profile for the PROLI NONOate treated and control membrane module. The TMP values are the daily average transmembrane pressures. The vertical dashed line splits the TMP profile into the low pressure phase (left side) and the rapid TMP increase phase (right side).


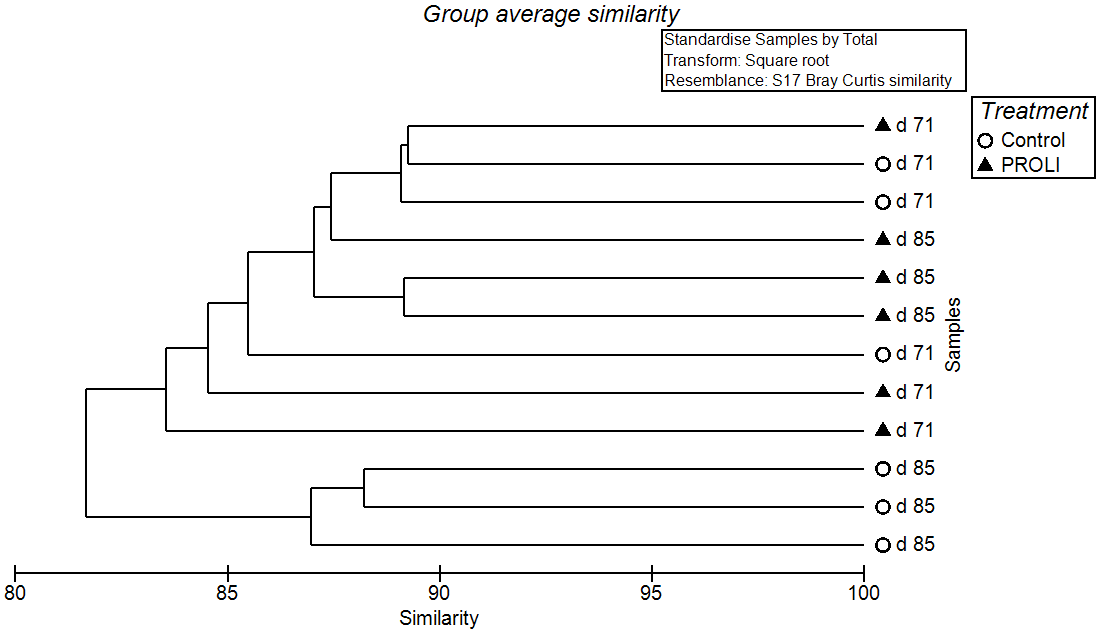


**Fig. S2.** The clustering tree of the bacterial communities for the PROLI NONOate treated and control biofilms at 71 and 85 d. The clustering tree was constructed based on the average Bray-Curtis similarity. The labels “Control” and “PROLI” represent the control biofilms and PROLI NONOate treated biofilms respectively.


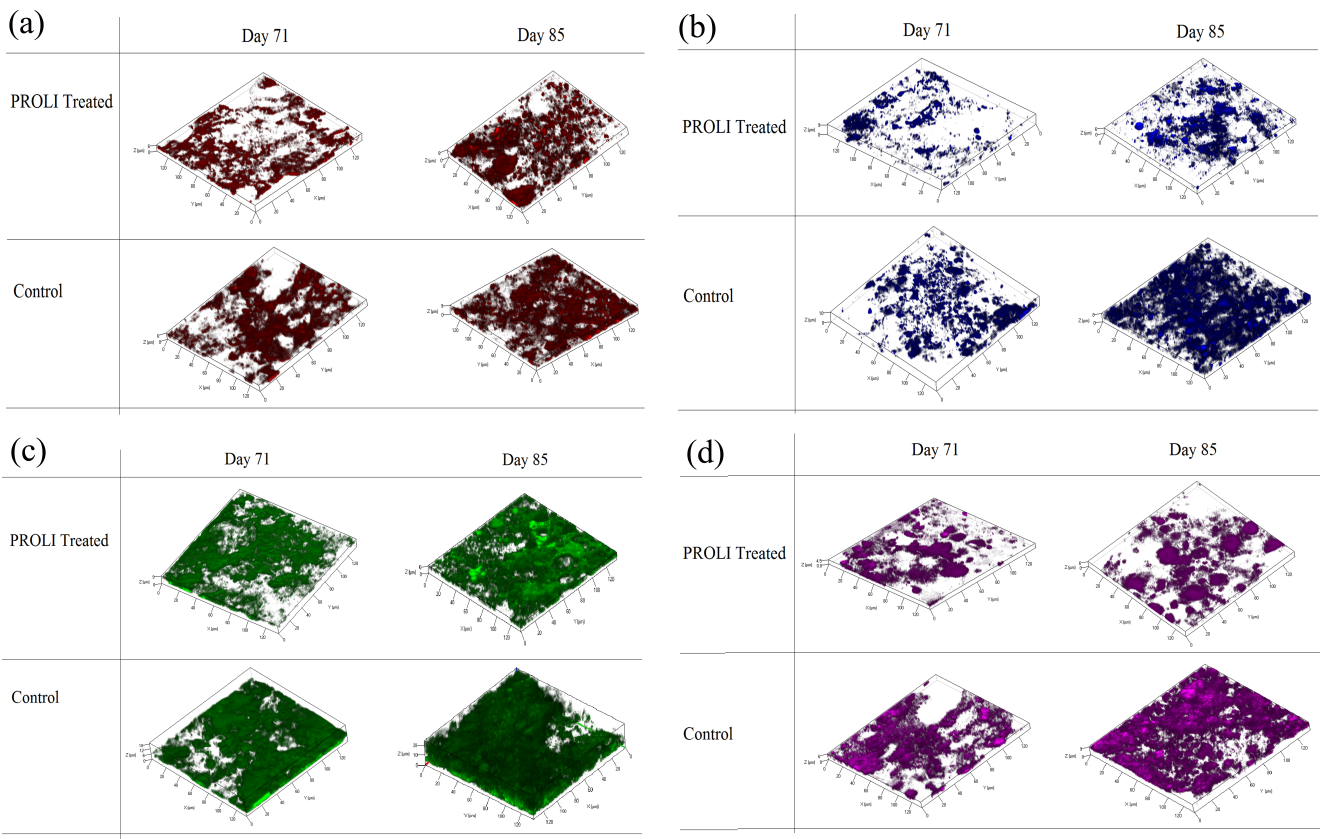


**Fig. S3.** Representative 3D confocal laser scanning microscopic (CLSM) images of biofilm matrix components and cells on the PROLI NONOate treated and control membranes. The α-polysaccharides (a) are shown in red, the β-polysaccharides (b) are shown in blue, the proteins (c) are shown in green and the microorganisms (d) are shown in purple. All images are top down projects of 3-D reconstructions of the biofilm. The total magnification for the images was 630 ×.


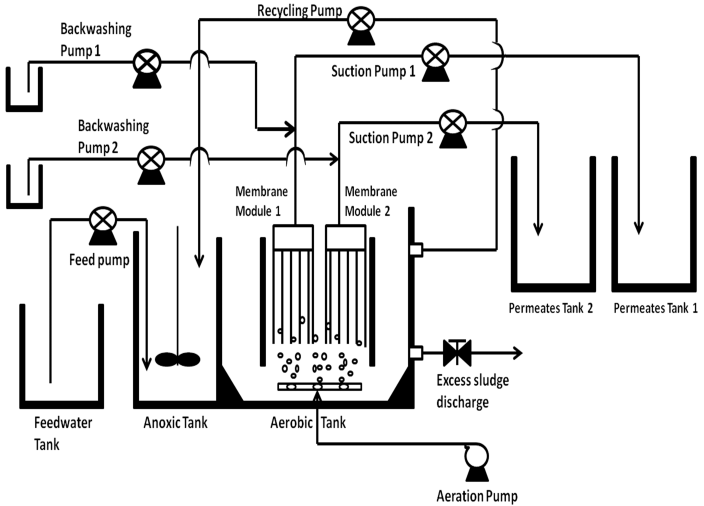


**Fig. S4.** Schematic drawing of the internal submerged MBR.

**Figure legends**

**Fig. S1.** The TMP increasing profile for the PROLI NONOate treated and control membrane module. The TMP values are the daily average transmembrane pressures during the operations of the MBRs. The vertical dashed line splits the TMP profile into two phases, after which the TMP of control membrane module increased faster and entered the jumping phase earlier than the TMP of PROLI NONOate treated membrane module.

**Fig. S2.** The clustering tree of the bacterial communities for the PROLI NONOate treated and control biofilms at 71 and 85 d. The clustering tree was constructed based on the average Bray-Curtis similarity. The labels “Control” and “PROLI” represent the control biofilms and PROLI NONOate treated biofilms respectively.

**Fig. S3.** Representative 3D confocal laser scanning microscopic (CLSM) images of biofilm matrix components and cells on the PROLI NONOate treated and control membranes. The α-polysaccharides (a) are shown in red, the β-polysaccharides (b) are shown in blue, the proteins (c) are shown in green and the microorganisms (d) are shown in purple. All images are top down projects of 3-D reconstructions of the biofilm. The total magnification for the images was 630 ×.

**Fig. S4.** Schematic drawing of the internal submerged MBR used in this project.
